# Nivolumab in combination with radiotherapy for metastatic esophageal neuroendocrine carcinoma after esophagectomy: a case report

**DOI:** 10.1186/s40792-021-01307-3

**Published:** 2021-10-01

**Authors:** Kuniyasu Takagi, Teppei Kamada, Yoshinobu Fuse, Wataru Kai, Junji Takahashi, Keigo Nakashima, Yuichi Nakaseko, Norihiko Suzuki, Masashi Yoshida, Shinya Okada, Hironori Ohdaira, Yutaka Suzuki

**Affiliations:** 1 Department of Surgery, International University of Health and Welfare Hospital, 537-3, Iguchi, Nasushiobara City, Tochigi 329-2763 Japan; 2 Department of Pathology, International University of Health and Welfare Hospital, Nasushiobara City, Japan

**Keywords:** Immune checkpoint inhibitors, Neuroendocrine carcinoma, Radiotherapy

## Abstract

**Background:**

Metastatic neuroendocrine carcinoma has an extremely poor prognosis, and no effective second-line treatment is available. Herein, we describe a case of multiple metastases after primary resection of esophageal neuroendocrine carcinoma successfully treated with nivolumab plus radiotherapy in a short time.

**Case presentation:**

A man in his 70s presented to our hospital after an abnormality was detected on an upper gastrointestinal series. Upper gastrointestinal endoscopy revealed a type 2 tumor spanning the endothelial cell junction to the abdominal esophagus. Histopathological examination of the biopsy confirmed a diagnosis of esophageal neuroendocrine carcinoma. The patient had no distant metastases. Thoracoscopic esophagectomy with three-field lymph node dissection was performed. Histopathological examination confirmed a diagnosis of esophageal neuroendocrine carcinoma with features of adenoid cystic-like carcinoma and squamoid pattern (pT2 [MP], INF a, ly1, v1 [EVG], pIM0, pDM0, pRM0, pN1 [1/28], M0; Stage II), which was positive for synaptophysin. The postoperative course was good, with no complications. The patient was treated with 100 mg of irinotecan and 100 mg of cisplatin, administered every 4 weeks, as postoperative adjuvant chemotherapy. Grade 3 loss of appetite was observed, and adjuvant chemotherapy was discontinued after four cycles of first-line treatment. A positron emission tomography–computed tomography scan 3 years after surgery showed abnormal uptake in the subaortic, left hilar, and left axillary lymph nodes, and in a mass in the right lung apex. The patient was diagnosed with metastatic esophageal neuroendocrine carcinoma postoperatively. First-line treatment could not be repeated due to toxicity from the initial treatment. Nivolumab (240 mg every 2 weeks) was administered as second-line treatment, and radiotherapy was started (56 Gy delivered in 28 fractions to the local [subaortic and hilar] lymph nodes). After 10 cycles of nivolumab in combination with radiotherapy (56 Gy), a positron emission tomography–computed tomography scan showed disappearance of all lesions. A complete response was achieved. Maintenance therapy (240 mg of nivolumab) was continued. No recurrence has been observed for 42 months.

**Conclusions:**

We experienced a case in which nivolumab in combination with radiotherapy was effective for metastatic esophageal neuroendocrine carcinoma after primary resection.

## Background

Esophageal neuroendocrine carcinomas (NECs) are rare tumors that account for approximately 0.26% of all malignancies of the esophagus. NECs exhibit a variety of morphological, functional, and behavioral characteristics that differentiate them from typical esophageal cancers, such as squamous cell carcinoma and adenocarcinoma. In a previous study [1], 30.3% of patients with NEC had lymph node metastases, and 48.7% had distant metastases, at initial diagnosis, with a median overall survival of 12 months. Therefore, the prognosis of patients with NEC is extremely poor.

Surgical resection with adjuvant chemotherapy is recommended for patients with resectable stage I–III esophageal NEC [2]. For treating NEC, the guidelines recommend platinum-based regimens, such as cisplatin plus irinotecan or etoposide, which are suitable for small-cell lung carcinoma (SCLC), regardless of the primary organ [3]. In general, regimens that have not been used as first-line treatment are used as second-line treatment. However, the optimal second-line treatment strategy for NEC remains to be determined.

Recently, the effectiveness of combination therapy with immune checkpoint inhibitors and radiotherapy for activating tumor immunity in non-SCLC and malignant melanoma has been demonstrated in a clinical trial [4]. However, to date, there have been no reports on the effectiveness of combination therapy with immune checkpoint inhibitors and radiotherapy for gastrointestinal NEC.

Herein, we describe a case of multiple metastases after primary resection of esophageal NEC successfully treated with nivolumab plus radiotherapy as second-line treatment in which in a complete response was achieved in a short period.

## Case presentation

**Fig. 1:**
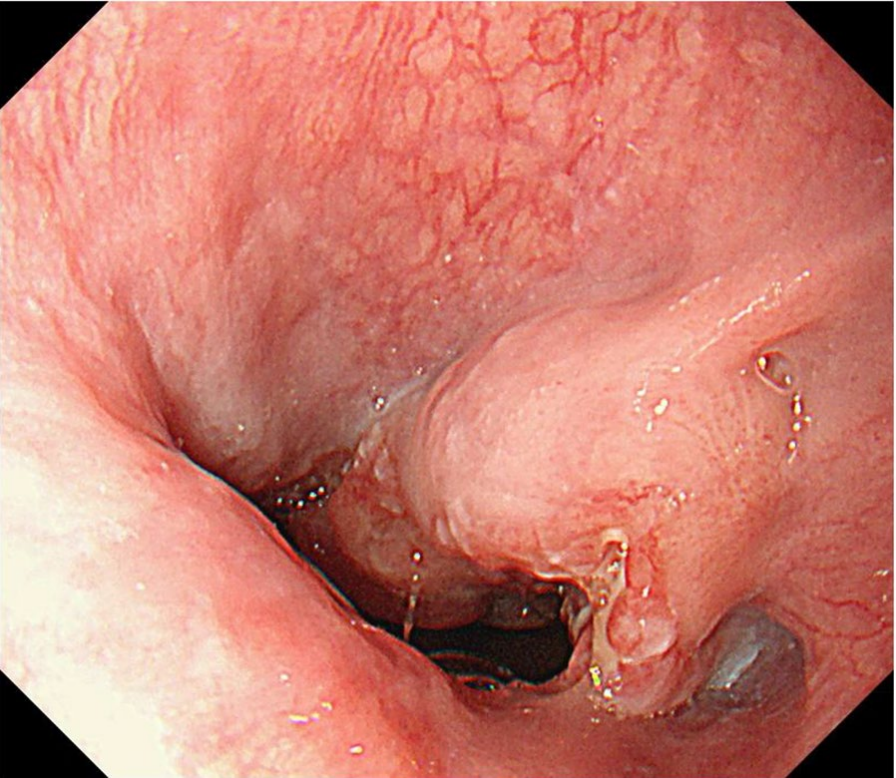
Preoperative upper gastrointestinal endoscopy. Endoscopy revealed a type 2 tumor spanning the endothelial cell junction to the abdominal esophagus

**Fig. 2:**
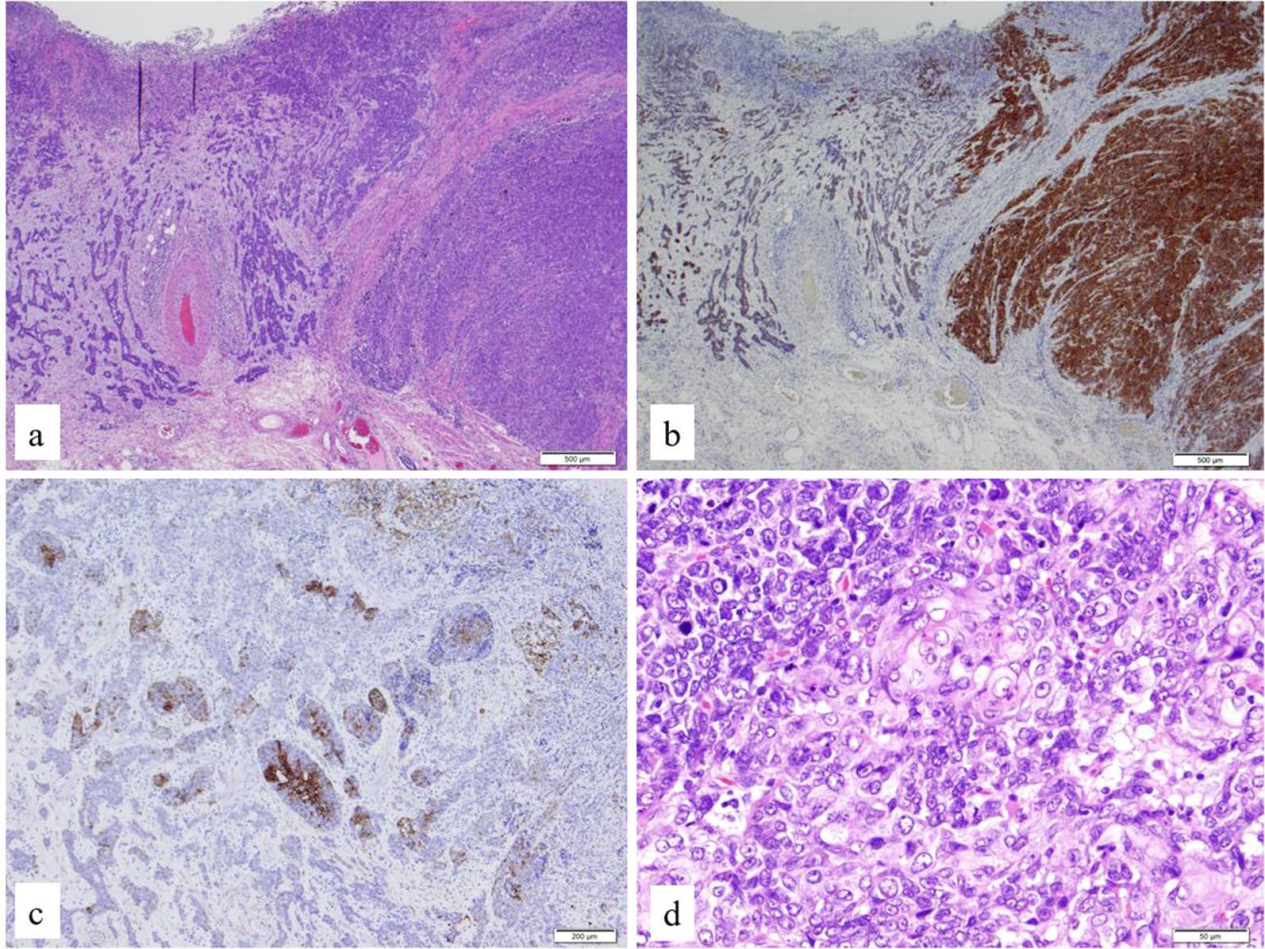
Histopathological analysis using hematoxylin and eosin and immunohistochemistry. **a** Esophageal neuroendocrine carcinoma with features of adenoid cystic-like carcinoma and squamoid pattern (magnification, × 4). **b** Tumor cells stained positive for synaptophysin (magnification, × 2). **c** Features of adenoid cystic-like carcinoma stained positive for cytokeratin 7 (magnification, × 4). **d** Features of squamoid pattern (magnification, × 20)

A man in his 70s presented to our hospital after an abnormality was detected on an upper gastrointestinal series. The patient had no significant medical or family history. The performance status was 0. The results of blood tests on admission were unremarkable, including tumor markers, such as carcinoembryonic antigen, carbohydrate antigen 19–9, and cytokeratin 19 fragment. Upper gastrointestinal endoscopy revealed a type 2 tumor (biopsy-confirmed NEC) spanning the endothelial cell junction to the abdominal esophagus (Fig. 1). Histopathological examination of the biopsy confirmed a diagnosis of esophageal NEC. Preoperative contrast-enhanced computed tomography (CT) and positron emission tomography (PET)-CT scans showed no distant metastasis, and esophagectomy was planned. Thoracoscopic esophagectomy with three-field lymph node dissection, gastric tube reconstruction, and feeding jejunostomy tube placement were performed. The operative time was 493 min, and the blood loss was 20 mL. Histopathological examination confirmed a diagnosis of esophageal NEC with features of adenoid cystic-like carcinoma and squamoid pattern (pT2 [MP], INF a, ly1, v1 [EVG], pIM0, pDM0, pRM0, pN1 [1/28], M0; Stage II), which was positive for synaptophysin. Immunohistochemistry revealed a Ki-67 labeling index of 3–30%, with 23 mitoses per high-power field. Tumor cells were positively stained for synaptophysin, CD56, neuron-specific enolase, P40, p53, and cytokeratin 7, and negatively stained for chromogranin A, cytokeratin 20, and CK20 (Fig. 2a–d). The positive rate of programmed death-ligand 1 (PD-L1) expression was ≤ 1%. The patient was wild type for the UGT1A1*6/*28 polymorphisms. The postoperative course was good, with no complications.

The patient was treated with 100 mg of irinotecan and 100 mg of cisplatin, administered every 4 weeks, as postoperative adjuvant chemotherapy. Grade 3 loss of appetite was observed, and adjuvant chemotherapy was discontinued after four cycles of first-line treatment. No recurrence was subsequently observed.

**Fig. 3:**
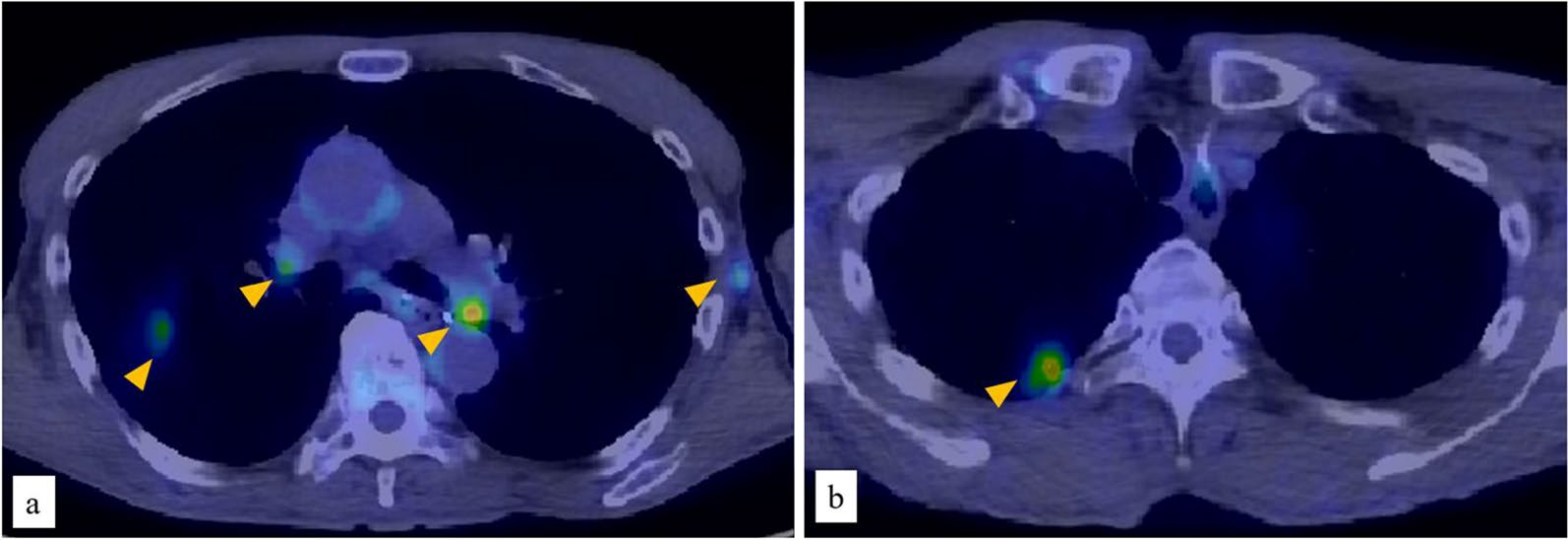
Positron emission tomography–computed tomography imaging at the delayed phase, showing the diagnosis of multiple metastases. **a** Abnormal uptake in the subaortic, left hilar, and left axillary lymph nodes (arrow heads). **b** Abnormal uptake in a mass in the right lung apex (arrow head)

**Fig. 4:**
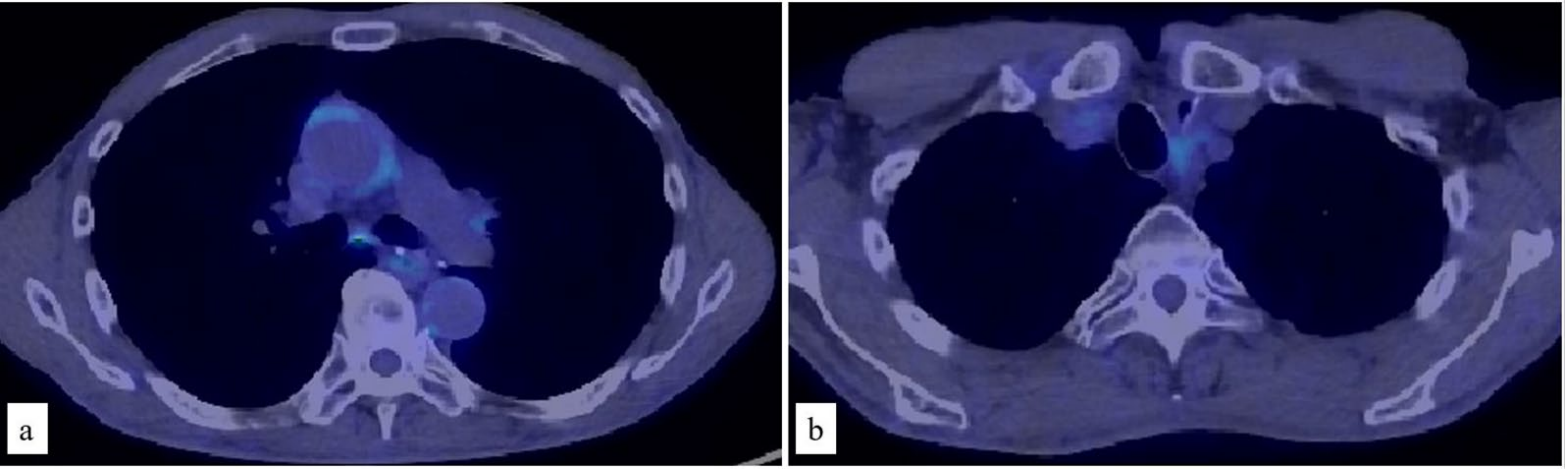
Positron emission tomography–computed tomography imaging at the delayed phase to evaluate the treatment response. **a**, **b** Disappearance of all lesions after 10 cycles of nivolumab in combination with radiotherapy (56 Gy)

A PET-CT scan 3 years after surgery showed abnormal uptake in the subaortic, left hilar, and left axillary lymph nodes, and in a mass in the right lung apex (Fig. 3a, b). The patient was diagnosed with metastatic esophageal NEC postoperatively. First-line treatment could not be repeated due to toxicity from the initial treatment. Nivolumab (240 mg every 2 weeks) was administered as second-line treatment, and radiotherapy was simultaneously started (56 Gy delivered in 28 fractions to the local [subaortic and hilar] lymph nodes). The protocol of the combination therapy for NEC was approved by the Institutional Review Board of International University of Health and Welfare Hospital (Approval No. 21-B-28), and the patient provided written informed consent. The only adverse event during treatment was Grade 1 myelosuppression. After 10 cycles of nivolumab in combination with radiotherapy (56 Gy), a PET-CT scan was performed to evaluate the treatment response according to the Response Evaluation Criteria in Solid Tumors (Version 1.1) [5]. The scan showed disappearance of all lesions, and a complete response was considered to have been achieved (Fig. 4a, b). Maintenance therapy (240 mg of nivolumab) was continued. No recurrence has been observed for 42 months.

## Discussion

We experienced a case in which a complete response was observed in a patient with metastatic esophageal NEC after surgery who was successfully treated with nivolumab in combination with radiotherapy. Combination therapy with immune checkpoint inhibitors and radiotherapy may be a valuable treatment option for NEC recurrence.

The prognosis of patients with gastrointestinal NEC is extremely poor. However, there are few reports on the prognosis and prognostic factors due to the rarity of the disease. Currently, only two large-scale multicenter retrospective studies have been published [6, 7]. Yamaguchi et al*.* [6] examined the outcomes of 258 patients with unresectable or recurrent gastrointestinal NEC in 23 multicenter retrospective studies. Primary sites included the esophagus (85 patients), stomach (70 patients), colorectum (31 patients), pancreas (31 patients), hepatobiliary system (31 patients), and small bowel (six patients). The most frequent metastatic sites were the liver (53%) and lymph nodes (51%). Irinotecan or etoposide plus cisplatin was administered as first-line chemotherapy in 80% of cases. The median overall survival of patients with the primary site in the esophagus, stomach, colorectum, pancreas, hepatobiliary system, and small bowel was 13.4, 13.3, 7.6, 8.5, 7.9, and 29.7 months, respectively. Each primary site was associated with an extremely poor prognosis. The authors concluded that a high-quality randomized controlled trial is required to determine the optimal treatment strategy [6]. Sorbye et al*.* [7] also examined the outcomes of 305 patients with advanced gastrointestinal NEC in 12 multicenter retrospective studies. Palliative chemotherapy was administered to 252 patients (cisplatin plus etoposide [51%], carboplatin plus etoposide [27%], carboplatin plus etoposide or vincristine [11%], and other drugs [11%]), while the remaining 53 patients received best supportive care. The median overall survival of the 252 patients treated with chemotherapy was 11 months, compared to 1 month for the 53 patients receiving best supportive care. The response rate to first-line treatment was just 31%. The authors concluded that patients with advanced gastrointestinal NEC should be treated with chemotherapy without delay due to its poor prognosis [7].

The 2016 European Neuroendocrine Tumor Society consensus guidelines [8] recommend platinum-based regimens, such as cisplatin plus irinotecan or etoposide (which are suitable for SCLC regardless of the primary organ), as first-line treatment for gastrointestinal NEC. Noda et al*.* [3] conducted a multicenter, randomized, phase III trial in which they compared irinotecan plus cisplatin with etoposide plus cisplatin in patients with SCLC. The median survival was longer in the irinotecan group than in the etoposide group (12.8 vs. 9.4 months, respectively; log-rank *p* = 0.002). The authors concluded that irinotecan plus cisplatin was effective for the treatment of metastatic SCLC [3]. However, the response rate to first-line treatment was still low [7, 8], and there was no recommendation for second-line treatment [8]. In a previous study [6], amrubicin was the most common second-line treatment. However, the response rate was only 4%, and the progression-free survival rate was only 1.9% [6].

There is a lack of evidence supporting the efficacy of immune checkpoint inhibitors alone for NEC. However, nivolumab has been shown to be effective for SCLC [9], and it may also be effective for NEC, which is typically treated similarly to SCLC. In addition, there is no clear evidence for radiation therapy alone for NEC, although it has been reported to be effective in symptomatic palliation and suppression of local progression. [10] In recent years, it has come to be thought that the combination of immune checkpoint inhibitors and radiation therapy will maximize their individual therapeutic effects for the treatment for various malignancies.

The effectiveness of combination therapy with immune checkpoint inhibitors and radiotherapy for activating tumor immunity in non-SCLC and malignant melanoma has been reported [4]. A clinical trial (NCT03544736) is currently underway to determine the efficacy of nivolumab in combination with radiotherapy for esophageal cancer. The mechanism of combination treatment with immune checkpoint inhibitors and radiotherapy may be explained as follows. Radiation-induced DNA and cell membrane damage, and the production of cytoplasmic reactive oxygen species, activates a variety of transcription factors and signaling pathways that modulate the immunophenotype and immunogenicity of tumor cells. Moreover, irradiation increases antigen presentation by enhancing surface MHC class I expression. This, in turn, induces phagocytosis and immunity by enhancing surface calreticulin expression and HMGB1 release, and apoptosis by increasing membrane Fas ligand expression. Irradiation-induced tumor cell disruption also stimulates dendritic cells by releasing damage-associated molecular patterns, activating cell-mediated immune responses, and enhancing tumor antigen cross-presentation [10]. The detailed mechanism(s) of combination treatment with immune checkpoint inhibitors and radiotherapy is still unknown. However, it is thought to involve the enhancement of the radiation-sensitizing effect and immune cell damage due to increased immune-induced cell death. The occurrence of systemic antitumor effects manifesting as regression of tumors outside of the irradiated field (abscopal effect) is also expected [11].

In the present case, nivolumab was selected, because the undifferentiated NEC exhibited features of adenoid cystic-like carcinoma and squamoid pattern, and immune checkpoint inhibitors are expected to be effective even for non-SCLC components. The patient did not have poor prognostic factors, such as a poor performance status, primary colorectal tumors, an elevated platelet count, or elevated lactate dehydrogenase levels [7], and nivolumab can be used regardless of tumor PD-L1 expression [12].

The optimal timing of immune checkpoint inhibitors and radiation therapy is still unclear. Nivolumab acts in the effector phase and inhibits the binding of PD-1 and PD-1 ligand to maintain T-cell activation and enhance anti-tumor immune effects; therefore, its simultaneous use with radiation is considered to be effective. While there are no studies that directly compare the timing of irradiation with immune checkpoint inhibitors, a study using mouse models reported that the effects of immune checkpoint inhibitors were diminished if administered more than 6 days after irradiation [13], suggested that the interval between immune checkpoint inhibitors and radiation therapy should not to be too long. In this case, all lesions, including axillary lymph node and lung lesions outside the irradiated field, disappeared. It was considered that the simultaneous combination of nivolumab and radiotherapy had caused the abscopal effect.

It has been reported [14] that the combination of immune checkpoint inhibitors and radiotherapy does not increase the risk of adverse events compared to chemotherapy alone. In this case, no adverse events of Grade 3 or higher were observed during the course, and nivolumab was administered as maintenance therapy. However, late adverse events and long-term prognosis are still unknown. Future studies involving a larger number of patients are required.

## Conclusions

We experienced a case in which nivolumab in combination with radiotherapy was effective for metastatic esophageal NEC after primary resection.

## Data Availability

Data sharing is not applicable to this article as no data sets were generated or analyzed during the current study.

## Abbreviations

CT: Computed tomography
NEC: Neuroendocrine carcinoma
PD-L1: Programmed death-ligand 1
PET: Positron emission tomography
SCLC: Small-cell lung carcinoma
